# *Paeonia lactiflora* Root Extract and Its Components Reduce Biomarkers of Early Atherosclerosis via Anti-Inflammatory and Antioxidant Effects In Vitro and In Vivo

**DOI:** 10.3390/antiox10101507

**Published:** 2021-09-23

**Authors:** Min Jeong Kim, Hyun-Hee Kang, Yeung Jin Seo, Kyung-Min Kim, Young-Jun Kim, Sung Keun Jung

**Affiliations:** 1 School of Food Science and Biotechnology, Kyungpook National University, Daegu 41566, Korea; minjung8128@gmail.com; 2 Department of Food Science and Technology, Seoul National University of Science and Technology, Seoul 01811, Korea; khh266900@seoultech.ac.kr; 3 Division of Crop Research, Gyeongsangbuk-do Provincial Agricultural Research & Extension Services, Daegu 41404, Korea; francisc@korea.kr; 4 School of Applied Biosciences, Kyungpook National University, Daegu 41566, Korea; kkm@knu.ac.kr; 5 Institute of Agricultural Science & Technology, Kyungpook National University, Daegu 41566, Korea

**Keywords:** *Paeonia lactiflora*, atherosclerosis, monocyte adhesion, vascular inflammation, VCAM-1, NF-κB

## Abstract

Although various physiological activities of compounds obtained from *Paeonia lactiflora* have been reported, the effects of *P. lactiflora* extract (PLE) on early atherosclerosis remain unclear. Therefore, in this study, we investigated the in vitro and in vivo antiatherosclerosis and in vitro antioxidant effects of PLE and its compounds. PLE suppresses the tumor necrosis factor (TNF)-α-induced capacity of THP-1 cells to adhere to human umbilical vein endothelial cells (HUVECs), vascular cell adhesion molecule (VCAM)-1 expression, and nuclear factor kappa-light-chain-enhancer of activated B cells (NF-κB) signaling in HUVECs. PLE also suppresses TNF-α-induced nuclear translocation of NF-κB p65 from cytosol as well as the enhanced *TNFA* and C-C motif chemokine ligand 2 (CCL2) mRNA expression in HUVECs. We identified and quantified the following PLE compounds using high-performance liquid chromatography with diode array detection: methyl gallate, oxypaeoniflorin, catechin, albiflorin, paeoniflorin, benzoic acid, benzoylpaeoniflorin, and paeonol. Among these, methyl gallate had the strongest inhibitory effect on monocyte adherence to TNF-α-induced HUVECs and the VCAM-1 expression. Reverse transcriptase real-time quantitative polymerase chain reaction showed that PLE compounds had a dissimilar inhibition effect on TNF-α-induced mRNA expression levels of *CCL2*, *TNFA*, and *IL6* in HUVECs. Except for paeonol, the compounds inhibited lipopolysaccharide (LPS)-induced reactive oxygen species production in RAW264.7 cells. In vivo, oral administration of PLE improved TNF-α-induced macrophage infiltration to the vascular endothelium and expression of VCAM-1, as well as *IL6* and *TNFA* gene expression in the main artery of mice. PLE could be useful as a nutraceutical material against early atherosclerosis via the combined effects of its components.

## 1. Introduction

Cardiovascular disease (CVD) is the leading cause of mortality globally, causing an estimated 17.9 million deaths each year, and is expected to account for more than 23.6 million deaths per year by 2030 [1]. As the main underlying pathology of CVD, atherosclerosis is caused by the chronic accumulation of vascular occlusion plaque in the endothelial layers of arteries, and it eventually results in severe stenosis that limits blood flow and causes significant tissue hypoxia [2]. Atherogenesis, which is the development process of atherosclerosis, can be defined by four steps in the lesion development process: initiation, promotion, progression, and acute events [3].

Low-density lipoprotein (LDL) on the inner artery promotes monocyte infiltration into the intima, also known as an “early atherosclerosis event” [4]. Once differentiated and activated, macrophages absorb excessive oxidized LDL (ox-LDL) and subsequently transform into lipid-laden foam cells [5]. Once this occurs, it is difficult to control the progression of atherosclerosis. Therefore, prevention of the early atherosclerosis event through consumption of nutraceuticals is a promising strategy.

The excessive production of proinflammatory cytokines, including tumor necrosis factor-alpha (TNF-α), interleukin 6 (IL-6), and monocyte chemoattractant protein-1 (MCP-1)/C-C motif chemokine ligand 2 (CCL2), is involved in the disruption of vascular homeostasis. Among them, TNF-α is an important proinflammatory cytokine that triggers vascular inflammation via activation of the NF-κB signaling pathways. Once NF-κB is dissociated from IκB, the inhibitor of NF-κB, it translocates from the cytosol to the nucleus and binds to the specific promoter region and subsequently to transcript vascular cell adhesion molecules and inflammatory cytokines [6]. Vascular cell adhesion molecule 1 (VCAM-1)/cluster of differentiation 106 (CD106) in the inflamed aortic endothelium contributes to the leukocyte extravasation and subsequent vascular inflammation in vitro and in vivo [7].

Reactive oxygen species (ROS) play a key role in endothelial dysfunction and atherogenesis [8]. Multiple studies have shown that antioxidant materials have a protective effect on vascular endothelial cells [9,10]. In the initiation stage of atherogenesis, the modification of LDL to ox-LDL in the presence of ROS is a key step, because macrophages recognize ox-LDL as nonself and start phagocytosis, a prerequisite for foam cell formation. Thus, the neutralization of excessive ROS is vital to control the pathogenesis of atherosclerosis.

*Paeonia lactiflora* Pall. (PLP) is well known in traditional medicine for its anti-inflammatory effects, including the inhibition of macrophage activation and regulation of immune cells or autoimmune diseases [11]. PLP is divided into shoot and root systems, and its root has long been used as traditional medicine in Korea, China, and other South Asian countries. The root contains several bioactive components, such as albiflorin [12], paeoniflorin [13], paeonol [14], and phenolic compounds, which can be modified by several processing steps for application as nutraceuticals and functional foods [15]. Parker et al. and Jiang et al. also have reported the pharmacological potential of bioactive constituents of *P. lactiflora* and *P. veitchii* Lynch [16] and total glucosides of paeony [17], respectively. Previous studies have demonstrated the positive effects of *P. lactiflora* components, including glycoside and paeoniflorin [18]. However, the evaluation of the *P. lactiflora* extract (PLE) as a functional food material for the treatment of early atherosclerosis remains unclear.

In this study, we studied the effect of PLE on the TNF-α-mediated infiltration of monocytes to the vascular endothelium by inhibition of VCAM-1 expression via regulation of NF-κB signaling pathways in human umbilical vein endothelial cells (HUVECs) and the effect of the oral administration of PLE on TNF-α-induced macrophage infiltration and production of proinflammatory cytokines in vivo. We also quantified eight compounds obtained from freeze-dried PLE using HPLC analysis and evaluated not only the effect of these compounds on TNF-α-mediated infiltration of monocytes to the vascular endothelium in HUVECs but also their effect on LPS-induced excessive ROS production in RAW264.7 cells to compare them. Our findings may help elucidate the preventive mechanism of PLE for early atherosclerosis.

## 2. Materials and Methods

### 2.1. Materials

Hyclone (Logan, UT, USA) provided Dulbecco’s modified Eagle’s medium (DMEM), RPMI 1640 medium, and fetal bovine serum (FBS). We obtained EBM-2 medium and EGM-2 Endothelial SingleQuots Kit from Lonza (Basel, Switzerland) and 2-mercaptoethanol from Gibco (Grand Island, NY, USA). We purchased recombinant human TNF-α from BD Pharmingen (San Diego, CA, USA) and recombinant mouse TNF-α from NKMAX (Seongnam-si, Gyeonggi-do, Korea). Sigma-Aldrich (St. Louis, MO, USA) provided 2′,7′-dichlorofluorescein diacetate (DCFH-DA) and analytical-grade reference standards (methyl gallate, catechin hydrate, paeoniflorin, benzoic acid, and paeonol). We used horseradish peroxidase (HRP)-conjugated pierce goat antirabbit IgG (H+L), pierce goat antimouse IgG (H+L), and Alexa Fluor 488 and 594 goat antirabbit IgG (H+L) for the secondary antibodies, purchasing these from Thermo Fisher Scientific Inc. (Eugene, OR, USA). The PLE reference standards, oxypaeoflorin, benzoyl paeoniflorin, and albiflorin, were purchased from Ensol Biosciences Inc. (Daejeon, Korea), Biosynth Carbosynth (San Diego, CA, USA), and FUJIFILM Wako Pure Chemical Corporation (Richmond, VA, USA).

### 2.2. Sample Preparation and Extraction Procedures

*P. lactiflora* root was obtained from the Bonghwa Herbal Crop Research Institue, GBARES, Bonghwa, Korea, in February 2019. The specimen was identified by a botanist, Young Jin Seo. The dried material was pulverized and then passed through an 8-mesh sieve. The powdered material (10 g) was extracted at 80% ethanol in a shaking incubator at room temperature for 24 h and a solvent-to-sample ratio of 50 to 1. The extract was filtered through quantitative filter papers (GVS, Zola Predosa, Bologna, Italy) before evaporation. The extract was then concentrated by rotary evaporation to eliminate ethanol. Then, the extract was freeze-dried in the range of 0.4–0.8 torr below –35 °C for 46 h and then stored at −20 °C until use. We prepared bamboo (*Phyllostachys pubescens*) leaf extracts as a positive control as described previously [19] and diluted them with distilled water immediately before use.

### 2.3. Cell Culture

We purchased human umbilical vein endothelial cells (HUVECs) and THP-1 cells, a human monocytic cell line, from Lonza (Basel, Switzerland), along with RAW264.7 cells, a mouse macrophage, from the Korean Cell Line Bank (KCLB; Seoul, Korea). We maintained HUVECs in an EBM-2 medium containing EGM-2 Endothelial SingleQuots Kit, passaged at 70−80 confluence, and cultured until passage 10. We maintained THP-1 in an RPMI 1640 medium supplemented with 10% FBS and 0.1% 2-mercaptoethanol; THP-1 cells were passaged between 2–6 × 10^5^ cells/mL. We maintained the RAW264.7 cells in DMEM, including 10% FBS; these cells were passaged at 70−80 confluence. We incubated all cells at 37 °C and 5% CO_2_ in a CO_2_ incubator (Thermo Fisher Scientific, Waltham, MA, USA).

### 2.4. Monocyte–Endothelial Cell Adhesion Assay

We performed the monocyte–endothelial cell adhesion assay as previously described [20]. We seeded the HUVECs at 1.5 × 10^5^ cells/mL into 96-well plates and incubated overnight to reach confluent monolayers. PLE and each compound were prepared with dimethyl sulfoxide (DMSO) as a stock solution. DMSO was not used at a final concentration of >0.1% (*v*/*v*) and was stored at −20 °C until use. We treated the HUVECs with 25, 50, and 100 μg/mL of PLE or compounds (50 μM) for 1 h and then stimulated them with 10 ng/mL TNF-α for 5 h. We prepared the fluorescently labeled THP-1 cells to be added to the activated HUVECs using 2 μM calcein AM (Sigma-Aldrich, St. Louis, MO, USA) for 15 min at 37 °C in phosphate-buffered saline (PBS). After adding the THP-1 cells to the HUVECs, we incubated these for 1 h, washing out the unbound THP-1 cells with PBS three times and measuring the monocyte adhesion at Ex = 485 and Em = 538 nm using a fluorescent plate reader (SpectraMax; Molecular Devices Corporation, Sunnyvale, CA, USA). Parthenolide was used as a positive control because it is a well-known agent to assess the anti-inflammatory effect via inhibition of NF-ĸB activation.

### 2.5. Western Blot Assay

We seeded the HUVECs at 5 × 10^4^ cells/mL and RAW264.7 cells at 3 × 10^5^ cells/mL in 100 mm dishes and cultured these for 24 h. We pretreated the cells with PLE with various concentrations (25, 50, and 100 μg/mL) or compounds (50 μM) for 1 h and then treated them with TNF-α (10 ng/mL) or LPS (1 μg/mL). We washed the cells twice using ice-cold PBS, scraped them with the cell lysis buffer (Cell Signaling Technologies, Beverly, MA, USA) containing protease and phosphatase inhibitor (Thermo Fisher Scientific), and then collected them. After 30 min, we centrifuged the collected cells at 13,000× *g* for 15 min. We then transferred the cell lysates into prechilled tubes and used these for the Western blot assay.

For protein quantification, we determined the cell lysate by reference to a standard curve of bovine serum albumin. We measured 30 μg of lysate protein, using the DC protein assay kit (Bio-rad Inc., Hercules, CA, USA). We separated the proteins with 10% sodium dodecyl sulfate–polyacrylamide gel (SDS−PAGE) electrophoretically and then transferred these to a polyvinylidene difluoride (PVDF) membrane (Millipore, Temecula, CA, USA). We blocked the transferred proteins using TBST containing 5% skim milk (BD biosciences) for 1 h at room temperature. After blocking, we incubated each membrane with specific primary antibodies overnight at 4 °C. After washing three times with TBST, we incubated horseradish peroxidase (HRP)-conjugated secondary antibodies against the species of primary antibodies for 1 h at room temperature. We detected the protein bands using a chemiluminescence detection kit (ATTO, Tokyo, Japan) and visualized the bands using GeneGnome XRQ NPC (Syngene, Cambridge, UK). We immunoblotted the samples for p-p65 (Ser536), p65, IκBα, p-IκB kinase (IKKα/β) (Ser176/180), IKKα, IKKβ, α/β-tubulin, HO-1, Keap-1, Nrf2 (1:1000, Cell Signaling Technologies, Beverly, MA, USA), anti-lamin B1 (1:10,000, Abcam, Cambridge, MA, UK), and VCAM-1 (1:1000, Santa Cruz Biotechnology, Santa Cruz, CA, USA).

### 2.6. Separation of Cytoplasmic and Nuclear Fractions

We seeded the HUVECs at 5 × 10^4^ cells/mL in 100 mm dishes and cultured them for 24 h. We pretreated the cells with PLE for 1 h and then treated them with TNF-α (10 ng/mL) for 15 min. We washed the cells twice with PBS and then fractionated them using NE-PER Nuclear and Cytoplasmic Extraction Reagents (Thermo Fisher Scientific) according to the manufacturer’s manual. We determined the separated cytoplasmic and nuclear proteins using Western blot assay.

### 2.7. Animals and Experiment Design

Central Lab Animal Inc. (Seoul, Korea) supplied 10-week-old male C57BL/6 mice (23−25 g). All mice were housed in a controlled environment with a 12 h light/dark cycle and maintained in cages in an air-conditioned room (23 ± 2 °C); we provided the mice free access to a standard chow diet and water. We randomly divided 25 mice into five groups (*n* = 5/group). In the control group (normal mice), we injected the mice intraperitoneally (i.p.) with PBS. In the TNF-α-treated group, we injected the mice i.p. with TNF-α at 25 μg/kg in PBS. We orally administered the extracts to the PLE low-dose (100 mg/kg body weight/day) treated group, PLE high-dose (500 mg/kg/day) treated group, and bamboo leaf extract (BLE; 100 mg/kg/day) treated group for 3 days. Then, we injected the mice i.p. with TNF-α daily for 7 consecutive days for early atherosclerosis 1 h after the last oral administration. At the end of 10 days, mice were anesthetized, and the aorta and heart were exposed and isolated.

### 2.8. Immunofluorescence

For the in vitro immunofluorescence (IF), we seeded the HUVECs at 2 × 10^4^ cells/mL in an 8-well-chamber slide glass and then cultured them overnight. We pretreated the cells with PLE for 1 h and then with TNF-α (10 ng/mL) for 15 min. We fixed the cells with 4% formaldehyde in PBS for 15 min after washing them with PBS. Then, we permeated the methanol into the cells at −20 °C for 15 min. After washing, we blocked the cells with a blocking buffer consisting of 5% FBS and 0.3% Tween 20 for 1 h at room temperature. We incubated the cells with specific primary antibodies in an antibody buffer overnight at 4 °C and then with goat polyclonal secondary antibody to rabbit IgG H&L (Alexa Fluor 488). We performed nuclear staining with a Vectashield antifade mounting medium with 4′,6-diamidino-2-phenylindole (DAPI) (Vector Laboratories, Burlingame, CA, USA), observed the cells under a fluorescence microscope, and recorded the images using LAS X (Leica Microsystems, Wetzlar, Hessen, Germany).

For the in vivo IF, we embedded the mouse heart and aorta in an optimal cutting temperature compound (FSC 22; Leica Microsystems, Lake, IL, USA), freezing and cryosectioning them. We obtained the histological data on 10 μm cryosections with a CM1850 cryostat (Leica Microsystems, Nussloch, Germany). We fixed the frozen tissue sections in 4% formaldehyde in PBS for 15 min, washed them three times with PBS, and blocked them with nonspecific binding using 5% FBS and 0.3% Tween 20 in PBS for 1 h at room temperature. We specifically stained the mouse aortic intimal surfaces using primary antibodies, including F4/80 and VCAM-1, overnight and washed the sections three times with PBS; then, we performed the same procedure as done with the in vitro IF protocol.

### 2.9. Quantitative Real-Time Polymerase Chain Reaction

We seeded the HUVECs at 5 × 10^4^ cells/mL in 100 mm dishes and cultured these for 24 h. We pretreated the cells with PLE or compounds (50 μM) for 1 h and then cultured them with TNF-α (10 ng/mL) for 24 h. We isolated the cells for RNA, using the RNAiso Plus (Takara Bio Inc., Kyoto, Japan) according to the manufacturer’s instructions. Then, we prepared the synthesis of the cDNA using the ReverTra Ace qPCR RT Master Mix (Toyobo, Osaka, Japan). We performed the real-time polymerase chain reaction (qRT-PCR) reactions with a CFX Connect real-time PCR detection system (Bio-rad) using SYBR Green real-time PCR master mix (Toyobo Co., Ltd., Osaka, Japan) according to the manufacturer’s manual. Table 1 describes the specific primers. We used the comparative Ct method for the data analyses using CFX Maestro Software (Bio-rad Inc.) and IBM SPSS 25.0 software package for Windows (SPSS Inc., Chicago, IL, USA), normalizing the values for each gene to *GAPDH* expression levels.

### 2.10. Dichlorofluorescein Diacetate Assay

We evaluated the ROS production by RAW264.7 cells exposed to LPS with a 2′,7′-dichlorofluorescein diacetate (DCF-DA) assay. We seeded the RAW264.7 cells at 2 × 10^5^ cells/mL in a 96-well plate overnight. We pretreated the cells with 50 μM of each compound in a 200 μL medium for 1 h. An effective ROS inhibitor, *N*-acetyl cysteine (NAC, 1 mM (32.64 μg/200 μL)), was used as a positive control. Subsequently, we stimulated the RAW264.7 cells by 1 μg/mL LPS for 24 h, washed them with PBS, and treated them with 20 μM of DCF-DA for 30 min in the dark. We then washed the cells twice with PBS. We measured ROS production at Ex = 485 and Em = 538 nm, using a fluorescent plate reader (SpectraMax; Molecular Devices Corporation, Sunnyvale, CA, USA).

### 2.11. Determination of PLE Component Content

We injected the samples using a high-performance liquid chromatography (HPLC) system (Agilent 1260 Infinity II Series, Santa Clara, CA, USA) equipped with a quaternary pump, an autosampler, and a diode array detector (DAD). A Unison Imtakt US-C18 column (250 × 4.6 mm, 5 μm) at 25 °C was used for the separation of the individual components of PLE. The reference standards, including methyl gallate, oxypaeoflorin, catechin, albiflorin, paeoniflorin, benzoic acid, benzoyl paeoniflorin, and paeonol, as well as PLE, were diluted with 70% ethanol. The range of the standard solutions was 0.4–50 µg/mL (0.4, 2, 10, 25 and 50 µg/mL). Then, 5 μL of the diluted standards and PLE were injected, and they were analyzed at a flow rate of 0.8 mL/min. We monitored the ultraviolet absorbance of the effluent at 240 nm. The HPLC mobile phase consisted of mobile phases A (0.01% phosphoric acid/water) and B (acetonitrile (ACN)). The gradient program was set as follows: 0 min, 5% B; 0–15 min, 5–45% B; 15–30 min, 45% B; 30–32 min, 45–5% B; 32–40 min, 5% B. The column was equilibrated with 70% ACN for 20 min at the end of the analysis, and it was confirmed that there were no residual analytes in the column.

The integrated peaks of PLE were quantified and identified by comparing their retention times against those of the corresponding standard peaks. All samples were analyzed by comparing the peak area with the external calibration curve from a neat standard solution. The content of each PLE component was estimated using Equation (1):

*P_spl_* = (*A_j_* − *B_o_*)/*B_1_* × *F*, (1)

where *P_spl_* is the concentration of each component, *A_j_* is the *j*th measurement of the area of the *i*th calibration standard, *B_1_* is the slope of the calibration curve, *B_o_* is the intercept of the calibration curve, and *F* is the dilution factor.

### 2.12. Statistical Analysis

We present the results as mean ± standard deviation (SD). We used the IBM SPSS 20.0 software package for the data analysis. We determined the significance of differences using a one-way analysis of variance considering the results significant at the 95% confidence level.

## 3. Results

### 3.1. PLE Ameliorates TNF-α-Mediated Monocyte Adhesion Capacity and Inhibits VCAM-1 and Inflammatory Cytokine mRNA Expression in HUVECs

**Figure 1:**
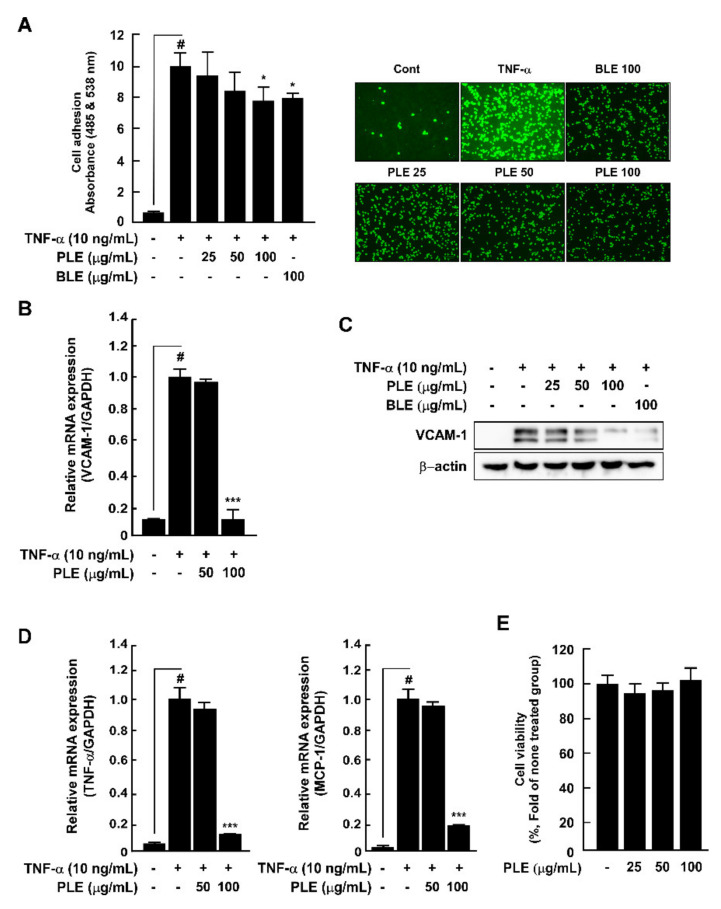
*Paeonia lactiflora* extract (PLE) ameliorated tumor necrosis factor (TNF-α)-induced monocyte adhesion capacity and inhibited vascular cell adhesion molecule (VCAM-1), TNF-α, and monocyte chemoattractant protein-1 (MCP-1) expression in human umbilical vein endothelial cells (HUVECs). (**A**) THP-1 cell adhesion to HUVECs after treatment with PLE and TNF-α. Fluorescently labeled THP-1 cells were assessed using fluorescent microscopy. (**B**) mRNA expression levels of *VCAM1* in HUVECs after PLE and TNF-α treatment for 24 h. (**C**) TNF-α-induced VCAM-1 expression in HUVECs after PLE and TNF-α treatment assessed using Western blotting. (**D**) *TNFA* and *CCL2* mRNA expression levels after PLE and TNF-α treatment assessed using qRT-PCR. (**E**) Cytotoxicity was evaluated using the MTT assay. # *p* < 0.05, compared with the control group; * *p* < 0.05 and *** *p* < 0.001, compared with the group exposed to TNF-α alone. Data are presented as the mean ± standard deviation of three independent experiments.

In the vascular system, the onset and development of inflammation are modulated by the expression of adhesion molecules, thereby locally recruiting monocytes to the endothelial cells [21]. In our study, we used BLE, as it is known to reduce vascular inflammation, as a positive control [19]. We found that PLE significantly suppressed TNF-α-induced monocyte THP-1 adhesion capacity on the HUVECs (Figure 1A). Activated endothelial cells express abnormal adhesion molecules, such as VCAM-1. PLE inhibited TNF-α-induced VCAM-1 expression in the HUVECs (Figure 1B,C). PLE diminished TNF-α-induced *TNFA* and *CCL2* (MCP-1) mRNA expression in the HUVECs (Figure 1D). PLE did not exhibit cell toxicity at any concentration (Figure 1E).

### 3.2. PLE Inhibits TNF-α-Induced NF-κB Signaling Regulator Phosphorylation and p65 Nuclear Translocation in HUVECs

**Figure 2:**
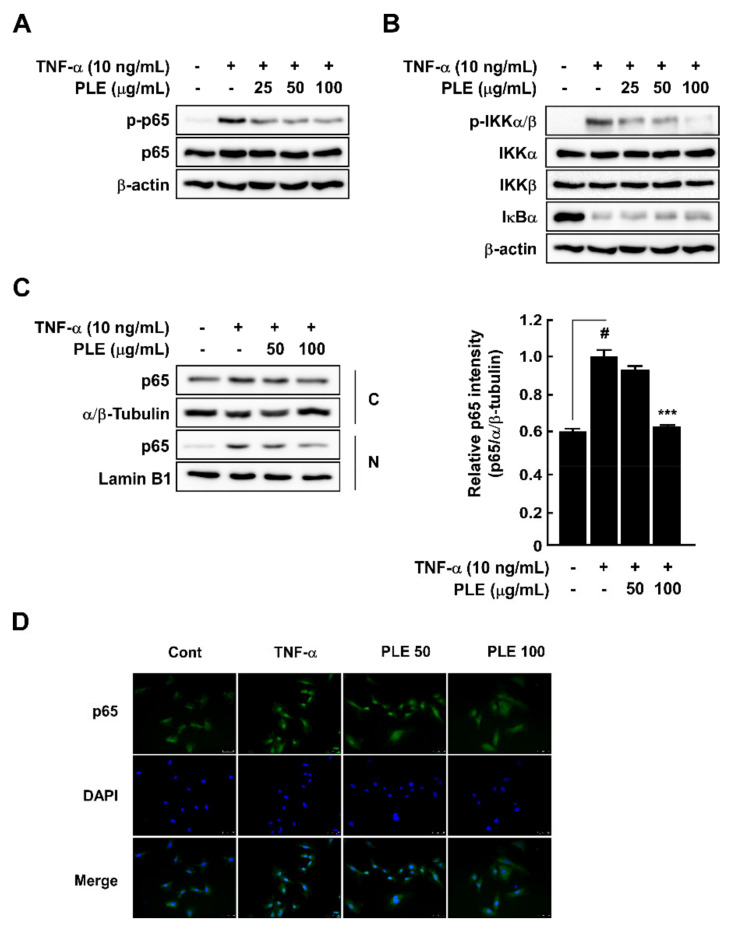
*Paeonia lactiflora* extract (PLE) rescued the TNF-α-induced NF-κB signaling pathway and p65 translocation toward the nucleus in HUVECs. (**A**) p65 and p-p65 levels were measured using Western blotting. (**B**) Phosphorylated and total IκB kinase (IKK) and IκB degradation levels after PLE and TNF-α treatment measured using Western blotting. (**C**) p65 translocation from the cytosol to nuclear was measured after PLE and TNF-α treatments using Western blotting. α/β-tubulin was used as the cytosolic protein loading control, and Lamin B1 was used as the nucleic protein loading control. (**D**) p65 localization after treatment with PLE and TNF-α in HUVEC was measured using immunofluorescence. p65 is shown with green staining, and the nucleus is shown with blue staining. # *p* < 0.05, compared with the control group; *** *p* < 0.001, compared with the group exposed to TNF-α alone. Data are presented as the mean ± standard deviation of three independent experiments.

VCAM-1 expression is related to the NF-ĸB signaling pathway [22]. In this study, we confirmed the effect of PLE on the TNF-α-induced NF-ĸB signaling pathway as PLE suppressing the TNF-α-induced phosphorylated p65 in the HUVECs (Figure 2A). PLE inhibited the TNF-α-induced phosphorylation of IκB kinase (IKK) and IĸB degradation in the HUVECs (Figure 2B). Acute 15 min treatment with TNF-α induced the translocation of p65 from the cytosol to the nucleus by activation of the phosphoryl residue at Ser 536. Our Western blot assays on cytosolic and nuclear cellular fractionations (Figure 2C) and IF (Figure 2D) showed that PLE significantly inhibited the translocation of p65 in the HUVECs.

### 3.3. Quantification of the Major Compounds in PLE Using HPLC

**Figure 3:**
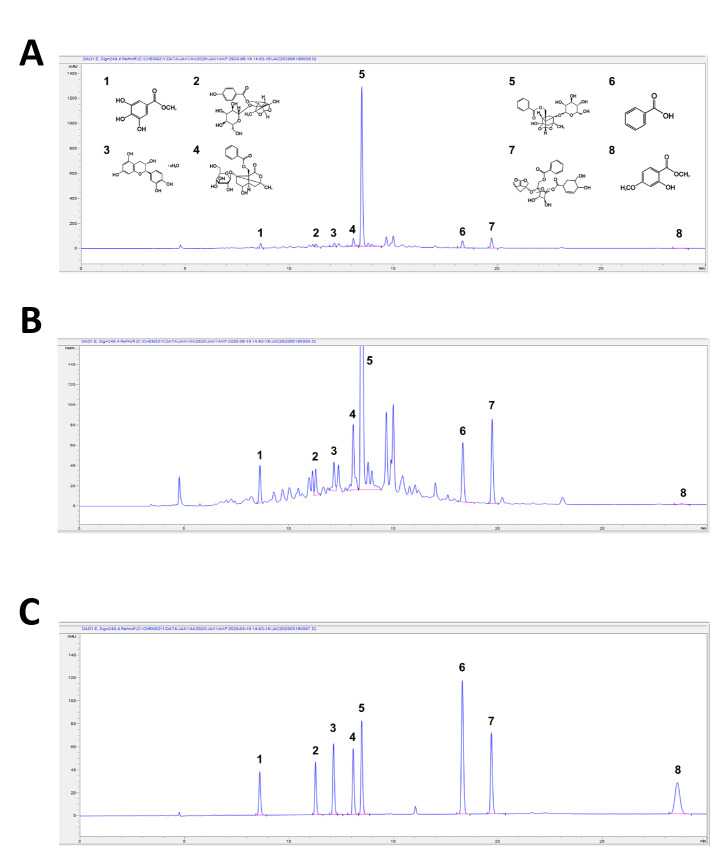
HPLC-UV (240 nm) profile of *Paeonia lactiflora* extract (PLE). (**A**,**B**) HPLC chromatogram of PLE. (**C**) HPLC chromatogram of PLE reference standards monitored at the wavelength of 240 nm. The identities of the compounds are as in Table 2 and the structures are in (**A**).

A major objective of this study was to identify each component in PLE using HPLC and to evaluate the efficacy of the corresponding substances; the PLE components were identified using reference standards. Figure 3A,B shows the HPLC chromatogram of root PLE. The HPLC chromatogram of the root PLE showed that the components of *P. lactiflora* were separated, with more than eight individual peaks, and their contents in accordance with reference standards and chromatogram of each reference are shown in Table 2 and Figure 3C, respectively.

Paeoniflorin showed the highest concentration at 83.47 mg/g, followed by benzoylpaeoniflorin (5.72 mg/g) and albiflorin (5.03 mg/g).

### 3.4. PLE Components Suppress TNF-α-Induced Monocyte Adhesion Capacity and Proinflammatory Cytokines in HUVECs

**Figure 4:**
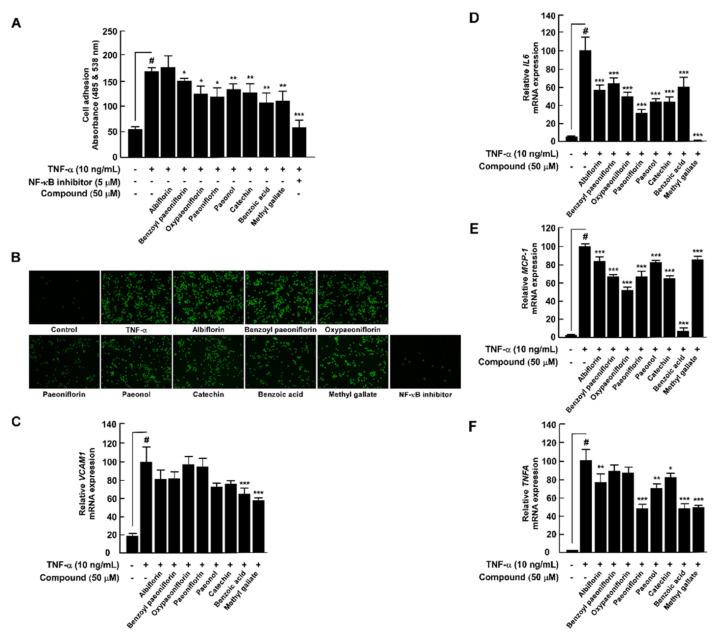
*Paeonia lactiflora* extract (PLE) compounds inhibit TNF-α-induced monocyte adhesion capacity and proinflammatory cytokines in HUVECs. (**A**) THP-1 cell adhesion to HUVECs after treatment with PLE components and TNF-α. Parthenolide (5 μM), an effective NF-ĸB inhibitor, was used as a positive control. (**B**) THP-1 cell adhesion to HUVECs was assessed using a fluorescence microscope. The relative mRNA levels of *VCAM1* (**C**), *IL6* (**D**), *CCL2* (**E**), and *TNFA* (**F**) were measured using qRT-PCR. # *p* < 0.05, compared with the control group; * *p* < 0.05, ** *p* < 0.01, and *** *p* < 0.001, compared with the group exposed to TNF-α alone. Data are presented as the mean ± standard deviation of three independent experiments.

We used each compound identified by HPLC to compare and evaluate whether it has an antiatherosclerotic effect. We performed this evaluation based on inhibiting the ability of the THP-1 cells to adhere to the TNF-α-induced HUVEC cells via inhibition of VCAM-1 expression in the HUVECs by the nutraceutical materials. Based on the inhibitory effects on leukocyte adherence to the HUVECs and VCAM-1 expression, we found that eight PLE components could prevent early atherosclerosis (Figure 4A–C). Benzoic acid (36.36%) showed the highest inhibitory potential, followed by catechin (35.78%), methyl gallate (34.36%), and paeoniflorin (29.71%). Dysregulated and excessive inflammatory response by the expression of cytokines such as IL-6 and TNF-α and chemokines including MCP-1 provokes vascular inflammation via overrecruiting migration and infiltration of the monocytes. PLE components significantly suppressed TNF-α-induced *IL6*, *TNFA*, and *CCL2* mRNA expression in the HUVECs (Figure 4D−F).

### 3.5. PLE Components Inhibit LPS-Induced ROS Production in RAW264.7 Cells

**Figure 5:**
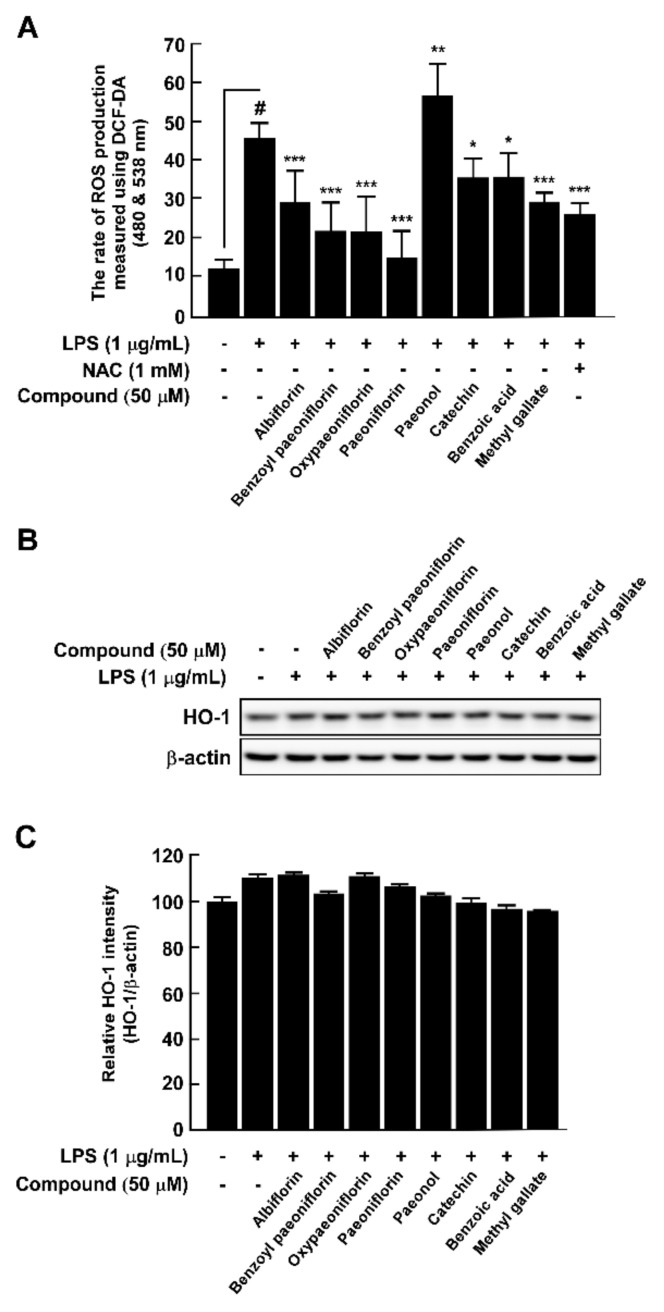
Antioxidant activity of *Paeonia lactiflora* extract (PLE) compounds against LPS-induced RAW264.7 cells. (**A**) Reactive oxygen species (ROS) production was determined using the 2′,7′-dichlorofluorescein diacetate (DCF-DA) method at 480 and 538 nm. An effective ROS inhibitor, *N*-acetyl cysteine (NAC, 1 mM), was used as a positive control. (**B**) HO-1 expression in RAW264.7 cells was measured using Western blotting. (**C**) HO-1 protein content was normalized to β-actin and quantified by ImageJ software. # *p* < 0.05, compared with the control group; * *p* < 0.05, ** *p* < 0.01, and *** *p* < 0.001, compared with the group exposed to LPS alone. Data are presented as the mean ± standard deviation of three independent experiments.

Oxidative stress associated with cellular damage by ROS plays a crucial role in early atherosclerosis [23]. Hence, we investigated whether PLE components suppressed ROS production in LPS-induced RAW264.7 cells, which have been used by other researchers to study oxidative stress in macrophages [24]. We used *N*-acetyl cysteine as the positive control because it acts as a scavenger of ROS. Except for paeonol, the PLE components significantly inhibited LPS-induced intracellular ROS generation (Figure 5A). Paeoniflorin (66.10%) is the most potent inhibitor, followed by oxypaeoniflorin (52.07%), benzyl paeoniflorin (51.78%), and methyl gallate (35.82%). However, the PLE components did not alter LPS-induced HO-1 expression (Figure 5B,C).

### 3.6. PLE Protects against TNF-α-Mediated Macrophage Infiltration toward the Aortic Root via Suppression of VCAM-1 and Expression of Proinflammatory Cytokines in the Mouse Aortic Arch and Aorta

**Figure 6:**
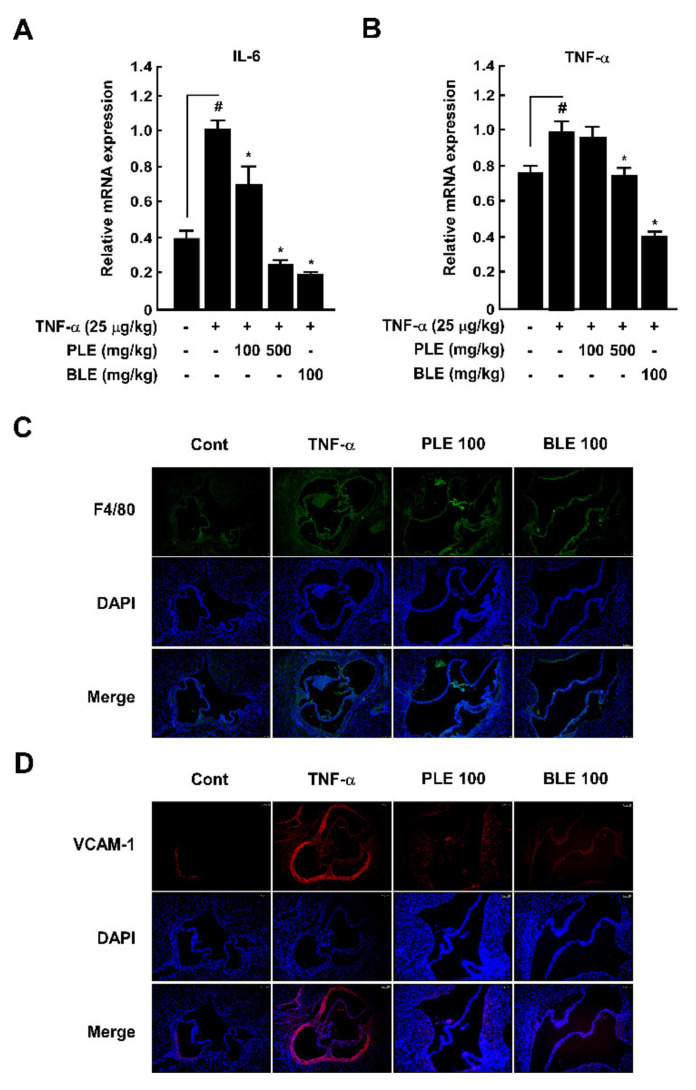
*Paeonia lactiflora* extract (PLE) rescued TNF-α-induced macrophage infiltration into the aortic root and suppressed *IL6* and *TNFA* mRNA expression in the aorta. The relative mRNA levels of *IL6* (**A**) and *TNFA* (**B**) were measured using qRT-PCR. Macrophage infiltration into the C57BL/6 mouse aortic root (**C**) and VCAM-1 expression (**D**) in the aorta of C57BL/6 mice following PLE and TNF-α treatment. # *p* < 0.05, compared with the control group; * *p* < 0.05, compared with the group exposed to TNF-α alone. Data are presented as the mean ± standard deviation of three independent experiments.

To ascertain the antiatherosclerotic effects of PLE, we investigated these effects against the infiltrated macrophages and enhanced VCAM-1 expression in the mouse aortic root using a TNF-α-induced mouse model of vascular inflammation. In our assessment, we considered proinflammatory cytokines as representative markers for early atherosclerosis. Therefore, to characterize the protective effects of PLE in vivo, we investigated TNF-α-induced expression of proinflammatory cytokines. Reverse transcriptase real-time quantitative polymerase chain reaction (qRT−PCR) showed that oral administration of PLE rescued TNF-α-mediated *IL6* and *TNFA* mRNA expression in the mouse aortic arch and aorta (Figure 6A,B). Oral administration of PLE inhibited TNF-α-mediated infiltrated macrophages in the intima of the aortic root (Figure 6C) and VCAM-1 expression in the aortic root (Figure 6D).

## 4. Discussion

CVD, the leading cause of death worldwide, is showing a slightly decreasing trend in developed countries but is still emerging as a social problem, with an increasing incidence rate in developing countries. Various statin treatments are prescribed for CVD, but patients with CVD do not develop symptoms until the final stage of clogged blood vessels, so treatment is limited.

In our previously published report, we classified atherogenesis, the development of atherosclerosis, as a major cause of CVD into initiation, promotion, progression, and acute events [3]. Therefore, we tried to develop nutraceuticals for the prevention of CVD. Among these stages, because the foam cell-mediated necrotic core is created in the progression stage and is difficult to control, we focused on the promotion stage and recruitment of monocytes and infiltration to the inner artery.

To develop nutraceuticals against atherogenesis, we have screened 20 species of botanical extracts; we selected PLE because it exhibited the greatest inhibitory effect on TNF-α-induced monocyte adhesion to vascular endothelial cells (data not shown). We reextracted in consideration of the in vivo experiment, performed the same experiment as before, confirmed that it showed similar efficacy, and then selected it as the final material.

Li et al. prepared total glycosides of paeony (TGP) capsules and confirmed their antiatherosclerotic effect in rats [25]. A recent study showed that a combination of total glucosides of *P. lactiflora* Pall. and total ligustici phenolic acids from *Ligusticum chuanxiong* Hort. has synergistic effects on focal cerebral ischemia in vitro and in vivo [18]. These data suggest that the intake of enhanced *P. lactiflora* components has a positive effect on vascular diseases such as atherosclerosis. However, these results did not confirm the activity of PLE or whether the promotion stage in atherogenesis was regulated.

We confirmed the preventive effect of PLE on TNF-α-induced atherogenesis by demonstrating the inhibition of the interaction between monocytes and vascular endothelium with similar contents of BLE and levels of *VCAM1* mRNA and protein expression in HUVECs. In our previous studies, *Lespedeza cuneata* extract and erythorbyl laurate were shown to exert antiatherosclerotic activities via inhibition of the interaction between HUVECs and THP-1 and VCAM-1 expression in vitro and in vivo [20,26]. These results indicate that PLE has protective properties for atherogenesis via the inhibition of monocyte adhesion to vascular endothelium. As the main signaling pathway to activate vascular inflammation, NF-κB plays a critical role in the gene expression of VCAM-1 and inflammatory cytokines including TNF-α and IL-6. As we expected, PLE suppressed the activity of TNF-α-induced p65, a subunit of NF-κB, translocation from the cytosol to the nucleus by suppressing phosphorylation of IKK and p65 in HUVECs.

G. You et al. confirmed that the content of Radix Paeoniae Alba, the dried root of *P. lactiflora* Pall., shows differences in chemical composition and antioxidant effects based on processing or excipient addition [27]. To evaluate the compound compositions of PLE and its effect on atherosclerotic markers, we identified and quantified PLE compounds using HPLC. A previous study demonstrated that TGP contains 15 monoterpene glycosides, including paeoniflorin, albiflorin, oxypaeoniflorin, benzoylpaeoniflorin, benzoyloxypeoniflorin, lactiflorin, albiflorin R1, benzoylalbiflorin, galloylpaeoniflorin, and paeoniflorin sulfonate [17]. Interestingly, using HPLC-DAD analysis, we found that the composition of PLE compounds is different from that of TGP components and newly identified gallate, catechin, and benzoic acid. As shown by the representative chromatograms of *P. lactiflora* root extract in Figure 3, paeoniflorin has a 15-fold higher concentration in PLE than the other compounds. In addition, when compared with the reported paeoniflorin content of *P. lactiflora* roots, the paeoniflorin content in *P*. *lactiflora* was >8%, which was higher than that of paeoniflorin, which is 2.0–4.0% in plants in the Paeoniaceae family [28]. Additionally, Yang Y. et al. identified that the paeoniflorin contents among the 20 *Paeonia* species ranged from 20.14 to 39.29 mg/g, which were lower than the paeoniflorin content of 83.47 mg/g in this study [29]. These variations in the phytochemical concentration of *P*. *lactiflora* are affected by regional changes, such as in temperature, soil, and rainfall, or seasonal changes, such as harvest time [30].

Although studies reported that paeonol and paeoniflorin are the main compounds in PLE that ameliorate vascular inflammation [31,32], few studies have evaluated the inhibitory effects of other PLE compounds on arteriosclerotic factors. We confirmed that not only paeonol and paeoniflorin but also other compounds showed inhibitory effects against the capacity of monocytes to adhere to the vascular endothelium. Among them, benzoic acid showed the highest inhibition capacity for THP-1 adhesion to HUVECs, followed by catechin and gallate. qRT-PCR results have also shown that gallate and benzoic acid had the highest inhibitory effects on TNF-α-induced *VCAM1* gene expression in HUVECs. Proinflammatory cytokines accelerate the influx and activation of inflammatory cells, such as differentiation from monocytes to macrophages [7,33]. Each PLE component had a distinct inhibition effect on TNF-α-induced *CCL2*, *TNFA*, and *IL6* mRNA expression in HUVECs. Benzoic acid showed the strongest inhibitory effect on *CCL2*; paeoniflorin, benzoic acid, and gallate showed the strongest inhibitory effects on *TNFA*; and gallate showed the strongest inhibitory effect on *IL6* mRNA expression in HUVECs. Benzoic acid, a plant phenolic acid derivative, is an antimicrobial preservative in food [34], but the effect against atherosclerosis-related vascular diseases is still unclear. Our study showed that benzoic acid could contribute to early atherosclerosis by inhibiting monocyte adhesion to the endothelium and abnormal *CCL2* mRNA expression. ROS also play a critical role in vascular inflammation and subsequent progression of atherogenesis [8]. Multiple studies have suggested that antioxidant botanical materials can be promising preventive antiatherosclerotic agents [35]. Paeoniflorin has the strongest antioxidant effect on LPS-induced ROS production in RAW264.7 cells. Additionally, except for paeonol, all PLE compounds exert antioxidant effects. These results indicate that even though paeoniflorin has the highest content among PLE compounds and exhibits ameliorating atherosclerosis factors, other ingredients also contribute to the control of vascular inflammation of PLE.

We conducted animal experiments to directly verify whether the in vitro efficacy is also shown in vivo. TNF-α as a stimulus in vivo can mediate endothelial activation such as aortic endothelial injury [36]. We already confirmed that intraperitoneal injection of TNF-α induced vascular inflammation, such as macrophage infiltration and abnormal mRNA expression of VCAM-1 and inflammatory cytokines such as IL-6 and TNF-α in the mouse aorta [37]. We found that oral administration of PLE significantly suppressed TNF-α-induced mRNA expression of proinflammatory cytokines, including *IL6* and *TNFA*, in the mouse aorta. Additionally, PLE blocked F4/80-positive monocyte infiltration into the intima and suppressed VCAM-1 expression in the mouse aortic root. These in vivo results proved that oral administration of PLE, not only single compounds, suppresses against the early stage of atherosclerosis via the regulation of monocyte infiltration into the vascular endothelium.

Collectively, we demonstrated that the protective effect of PLE is due to a combination of PLE compounds and does not only depend on specific compounds such as paeoniflorin and paeonol. Additionally, PLE has a protective effect on vascular inflammation, including monocyte infiltration to the inner artery and abnormal expression of cytokines, and has an antioxidant effect via inhibition of the NF-κB signaling pathways.

## 5. Conclusions

CVD, the world’s leading cause of death, is still emerging as a social problem. Because CVD does not develop symptoms until the final stage of clogged blood vessels, treatment for CVD is limited.

There is a lack of evidence on the effect of PLE against early atherosclerosis, a major cause of CVD. Thus, we have suggested that PLE suppresses TNF-α-induced monocyte adhesion to the vascular endothelium via suppressing the NF-κB pathway, including phosphorylation and nuclear translocation of p65.

We identified and quantified PLE compounds using HPLC. The PLE components identified using reference standards have shown different inhibitory effects on TNF-α-mediated *CCL2*, *TNFA*, and *IL6* mRNA expression and LPS-induced ROS production in the HUVECs and RAW264.7 cells, respectably. Thus, we demonstrated that the protective effect of PLE is due to a combination of PLE compounds and depends not only on specific compounds such as paeoniflorin and paeonol. Therefore, our findings demonstrated that PLE and PLE components are promising nutraceutical materials that can inhibit monocyte adherence to the monolayer of endothelial cells and lower the abnormal expression of proinflammatory cytokines.

## Figures and Tables

**Table 1:** List of gene-specific primer sequences.

	Gene	Gene Accession Number	Forward (5’–3’)	Reverse (5′–3′)
Human	*VCAM1*	NM_080682	ATGACATGCTTGAGCCAGG	GTGTCTCCTTCTTTGACACT
	*IL6*	NM_000600	TTCTCCACAAGCGCCTTCGGTCCA	AGGGCTGAGATGCCGTCGAGGATG
	*TNFA*	NM_000594	ATCAATCGGCCCGACTATCTC	GCAATGATCCCAAAGTAGACCTG
	*CCL2*	NM_002982	CTGCTCATAGCAGCCACCTT	CAGGTGACTGGGGCATTGAT
	*GAPDH*	NM_001357943	GAAGGTGAAGGTCGGAGTC	GAAGATGGTGATGGGATTTC
Mouse	*IL6*	NM_001314054	TGGGACTGATGCTGGTGACAAC	AGCCTCCGACTTGTGAAGTGGT
	*TNFA*	NM_001278601	TGGAACTGGCAGAAGAGGCACT	AGAGGCTGAGACATAGGCACCG
	*GAPDH*	NM_001289726	ACTCCACGACATACTCAGC	TCAACGGCACAGTCAAGG

**Table 2:** Individual compounds in root *Paeonia lactiflora* extract.

No.	Compound	Amount (mg/g)
1	Methyl gallate	4.796 ± 0.079
2	Oxypaeoniflorin	2.695 ± 0.034
3	Catechin	1.997 ± 0.038
4	Albiflorin	5.028 ± 0.149
5	Paeoniflorin	83.474 ± 1.004
6	Benzoic acid	2.479 ± 0.047
7	Benzoyl paeoniflorin	5.721 ± 0.097
8	Paeonol	0.079 ± 0.003

## Data Availability

The data presented in this study are available in article.
